# Non-specific and Usual Interstitial Pneumonia, Short-Term Survival After Surgical Biopsy

**DOI:** 10.1007/s00408-015-9721-y

**Published:** 2015-04-02

**Authors:** Barbara J. Knipscheer, Coline H. M. van Moorsel, Jan C. Grutters

**Affiliations:** 1 St. Antonius Hospital, Postbox 2500, 3420 EM Nieuwegein, The Netherlands; 2 University Medical Center Utrecht, Utrecht, The Netherlands

**To the Editors**,

Idiopathic interstitial pneumonia (IIP) is a diagnostic challenge. The diagnostic work-up in general includes medical history, physical examination, lung function tests, chest X-ray, high-resolution computed tomography and bronchoalveolar lavage. If no clinical diagnosis can be established on the basis of these data, a surgical lung biopsy might be indicated. In this setting, possible therapeutic and prognostic implications should be weighed against the risk of postoperative complications such as infections, prolonged air leakage, respiratory failure, acute exacerbation of underlying disease and even mortality.

Review of literature shows that postoperative mortality after surgical lung biopsy in IIP was found to be 4.8 % after 30 days and 6 % after 90 days [1]. Reported risk factors for mortality were low diffusing capacity for carbon monoxide (DLCO) and low forced vital capacity (FVC) [2, 3]. There are reports suggesting that surgical lung biopsy itself increases the risk of acute exacerbation of underlying disease [2, 3].

The aim of this study was to evaluate the 30- and 90-day mortality rate after surgical lung biopsy in two histological distinct groups of patients: one with a histological pattern of usual interstitial pneumonia (UIP) and one with a pattern of non-specific pneumonia (NSIP).

In this retrospective study, we included all patients with a histological UIP or NSIP who underwent surgical lung biopsy between 1993 and 2011 in our centre.

Baseline characteristics are presented in Table 1. Data on mortality rate at 30 and 90 days, length of postoperative hospital stay, duration of air leakage, time to drain removal after surgery and infectious complications were collected.

**Table 1:** *Baseline characteristics*

Characteristic	*N*	%	Median	*p* value
Gender (male/female)				0.007
UIP	35/11	76.1/23.9		
NSIP	10/13	43.5/56.5		
Smoking status (never/former/current)				0.160
UIP	13/22/8	30/51/19		
NSIP	12/7/3	54/32/14		
Age (years)				0.010
UIP	46		60.0	
NSIP	23		53.0	
BMI				0.248
UIP	40		28.0	
NSIP	21		27.4	
FEV1 (%)				0.004
UIP	40		84.0	
NSIP	19		66.0	
FVC (%)				0.233
UIP	34		74.0	
NSIP	16		70.0	
TLC (%)				0.143
UIP	21		72.0	
NSIP	13		68.0	
DLCO (%)				0.011
UIP	33		51.0	
NSIP	15		43.0	

Data of 69 patients were analysed (Table 1). There were 23 patients with histological NSIP and 46 with UIP. Baseline characteristics showed significant differences between both groups: mean forced expiratory volume in 1 s (FEV1) and diffusion capacity (DLCO) were lower in the NSIP group compared with the UIP group. Patients with a UIP pattern in this study were more often male and were older. No significant differences were found in FVC, total lung capacity, Body Mass Index (BMI) and smoking status.

Overall, the 90-day mortality rate after surgical lung biopsy in all patients was 11.6 % (8 out of 69).

In the UIP group, all patients who died underwent an open lung biopsy, the 30-day mortality rate was 8.7 % (4 out of 46) and the 90-day mortality rate was 15.2 % (7 out of 46). The median pre-operative FVC was 86 % and DLCO 46 % of the seven patients who passed away. Two patients died of pneumonia with radiologic evidence of progression of fibrosis. Post-mortem examination revealed diffuse alveolar damage in one case. Three patients died in the second and third month due to progression of fibrosis, and in two cases, this was proven by post-mortem examination and in one case radiologically. One cause of death was unknown due to loss to follow-up. Among seven patients with UIP who died, five were ultimately diagnosed to have idiopathic pulmonary fibrosis (IPF) and two to nonclassifiable interstitial pneumonia. In the NSIP group, only one patient died due to pneumonia in the first month after surgery. The 30- and 90-day mortality rates were both 4.3 % in the NSIP group.

No significant differences were found in postoperative hospital stay (*p* = 0.906), duration of air leakage (*p* = 0.721), time to drain removal after surgery (*p* = 0.65) and infectious complications (*p* = 0.977) between the NSIP and UIP group. There was no correlation between year of surgery and mortality.

Although the difference in mortality rate between the UIP and NSIP group was not statistically significant, we observed a trend towards a higher mortality rate in patients with UIP pattern, which is in agreement with reports in the literature [2, 3].

Interestingly, all deceased UIP patients with a known cause of death showed progression of fibrosis, while no progression of fibrosis was observed in the patient who died in the NSIP group. This observation suggests that the UIP non-survivors, who had relatively good pre-operative lung functions compared to the all NSIP patients, experienced rapid progression of their disease which cannot be excluded to be related to the surgical procedure.

We hypothesize that this rapid progression of fibrosis might be induced by the surgical procedure itself causing increased damage of the highly vulnerable alveolar epithelium. Increased vulnerability of the alveolar wall appears to be a fundamental problem in IPF.

In conclusion, mortality after surgical lung biopsy is relatively high, especially in patients with a histological pattern of UIP. We think that these results should be taken into account when considering open lung biopsy in patients with suspected IIP.
